# Insulin and Glucagon Impairments in Relation with Islet Cells Morphological Modifications Following Long Term Pancreatic Duct Ligation in the Rabbit – A Model of Non-insulin-dependent Diabete

**DOI:** 10.1155/EDR.2001.101

**Published:** 2001

**Authors:** J. Catala, M. Daumas, A. Pham Huu Chanh, B. Lasserre, E Hollande

**Affiliations:** 1 Laboratoire de Physiopathologie Cellulaire Université P. Sabatier Toulouse III 38, rue des 36 Ponts Toulouse 31400 France; 2 Laboratoire de Pharmacologie de la Régulation Université P. Sabatier Toulouse III 38, rue des 36 Ponts Toulouse 31400 France; 3 Laboratoire de Biotogie Cellulaire et Molécutaire des Epithéliums Université P. Sabatier Toulouse III 38, rue des 36 Ponts Toulouse 31400 France

## Abstract

Plasma levels of glucose, insulin and glucagon were measured at various time intervals after pancreatic duct ligation (PDL) in rabbits. Two hyperglycemic periods were observed: one between 15–90 days (peak at 30 days of 15.1 ± 1.2mmol/l, p < 0.01), and the other at 450 days (11.2 ± 0.5 mmol/l, p < 0.02). The first hyperglycemic episode was significantly correlated with both hypoinsulinemia (41.8 ± 8pmol/l, r= –0.94, p < 0.01) and hyperglucagonemia (232 ± 21ng/l, r=0.95, p < 0.01). However, the late hyperglycemic phase (450 days), which was not accompanied by hypoinsulinemia, was observed after the hyperglucagonemia (390 days) produced by abundant immunostained A-cells giving rise to a 3-fold increase in pancreatic glucagon stores. The insulin and glucagon responses to glucose loading at 180, 270 and 450 days reflected the insensitivity of B- and A-cells to glucose. The PDL rabbit model with chronic and severe glycemic disorders due to the predominant role of glucagon mimicked key features of the NIDDM syndrome secondary to exocrine disease.


Int. Jnl. Experimental Diab. Res., Vol. 2, pp. 101–112
Reprints available directly from the publisher
Photocopying permitted by license only

© 2001 OPA (Overseas Publishers Association) N.V.
Published by license under
the Harwood Academic Publishers imprint,
part of Gordon and Breach Publishing,
a member of the Taylor & Francis Group.
Printed in the U.S.A.

### Insulin and Glucagon Impairments in Relation with Islet Cells Morphological Modifications Following Long Term Pancreatic Duct Ligation in the Rabbit – A Model of Non-insulin-dependent Diabetes

J. CATALA[a,*], M. DAUMAS[a], A. PHAM HUU CHANH[b], B. LASSERRE[b] and E. HOLLANDE[c]

[a]*Laboratoire de Physiopathologie Cellulaire,* [b]*Laboratoire de Pharmacologie de la Régulation,*
[c]*Laboratoire de Biologie Cellulaire et Moléculaire des Epithéliums,*
*Université P. Sabatier, Toulouse III, 38, rue des 36 Ponts, 31400 Toulouse, France*

*(Received 16 August 2000; In final form 21 February 2001)*

**Plasma levels of glucose, insulin and glucagon were measured at various time intervals after pancreatic duct ligation (PDL) in rabbits. Two hyperglycemic periods were observed: one between 15–90 days (peak at 30 days of 15.1 ± 1.2 mmol/l, $p < 0.01$), and the other at 450 days (11.2 ± 0.5 mmol/l, $p < 0.02$). The first hyperglycemic episode was significantly correlated with both hypoinsulinemia (41.8 ± 8 pmol/l, $r = -0.94$, $p < 0.01$) and hyperglucagonemia (232 ± 21 ng/l, $r = 0.95$, $p < 0.01$). However, the late hyperglycemic phase (450 days), which was not accompanied by hypoinsulinemia, was observed after the hyperglucagonemia (390 days) produced by abundant immunostained A-cells giving rise to a 3-fold increase in pancreatic glucagon stores. The insulin and glucagon responses to glucose loading at 180, 270 and 450 days reflected the insensitivity of B- and A-cells to glucose. The PDL rabbit model with chronic and severe glycemic disorders due to the predominant role of glucagon mimicked key features of the NIDDM syndrome secondary to exocrine disease.**

*Keywords*: Rabbit; Pancreatic duct ligation (PDL); Insulin; Glucagon; Intravenous glucose tolerance test (IVGTT); Intravenous (i.v.); Immunocytochemistry; Non-insulin-dependent diabetes mellitus (NIDDM)

### INTRODUCTION

Modifications in islet cell mass are demonstrated to be largely responsible for the glycemic disturbances, and especially the hyperglycemia, in non-insulin-dependent diabetic (NIDDM) GK rats,[1] spontaneously diabetic Chinese hamsters[2] and secondary to pancreatitis.[3] or fibro-calculous-pancreatic diabetes (FCPD).[4] Over the past two decades, hyperglycemia-related hyperglucagonemia has also been observed in NIDDM patients,[5] streptozotocin rats[6] and alloxan-diabetic dogs,[7]

*Corresponding author. Tel./Fax: + 33(0)561 085 636, e-mail: catala@lmtg.ups-tlse.fr as well as in chronic pancreatitis.[8] Glucagon is found to cause the hyperglycemia in diabetic diseases by increasing hepatic glucose output (HGO), which stimulates gluconeogenesis in fasting NIDDM.[9] Moreover, the hyperglucagonemia of diabetes may be accounted for by defects in primary A- and B-cells[10] as well as by an impaired insulin secretion.

In a previous report,[11] we described the non-insulin-dependent diabetogenic effects following pancreatic duct ligation (PDL) in the rabbit.[11] The main short-term glycemic disorders were a transient hyperglycemia and glucose intolerance[11] concomitant with dissociation[12] and regeneration of the Langerhans islet cells during the first month.[13] The destruction of the islet architecture led to morphological and metabolic changes[14,15] notably at the time of minimum mass of B-cells (day 30). By the 5th and 35th days, the impairment in insulin secretion in the isolated perfused pancreas is characterized by loss of the early peak insulin release in response to glucose stimulation and the abolition of the potentiating effect of glucose on the arginine-induced secretion.[16] These effects resemble those noted in NIDDM,[17] FCPD,[18] acute pancreatitis,[19] and adult GK rats.[20]

The present study was designed to: (1) characterize glycemia and glucose tolerance after long-term pancreatic duct ligation, to discern relationships between glucose and insulin, glucose and/or glucagon in plasma, and correlations of plasma levels of these two hormones with pancreatic stores (2) study hormone regulation during regeneration and recasting of the endocrine parenchyma (3) compare this model system with NIDDM in humans and animals.

### MATERIALS AND METHODS

### Pancreatic Duct Ligation

Adult male rabbits (*Cuniculus oryctolagus*-provided by a licensed supplier) of 14–15 weeks, weighing 2.5 kg were fed with a commercial diet (U.F.A.C.-France) that contained 14% w/w protein. Food and water were available *ad libitum*. The rabbits were anesthetized with sodium pentobarbital 0.5 ml·kg body weight (Sanofi Health Animal Nutrition, 33501 Libourne cedex, France) by a single i.v. injection into the marginal vein of the ear. After laparatomy, the single duct was cut in between a double ligation placed at its opening into the duodenum, 40 cm from the pylorus in the PDL group, while the pancreas was simply handled in the control animals (C group).[21] All animal experiments have been approved by the authorities of the Ministry of Agriculture and Forestry: Direction of Health and Animal Protection (personal agreement n° 04411).

### Sampling and Analytical Procedures

Plasma glucose, insulin and glucagon concentrations were determined in both groups. The non-fasting rabbits were monitored at 0, 15, 30, 60, 90, 120, 180, 240, 340, 390 and 450 days after operation (20 C animals and 30 PDL animals). Blood samples were collected on EDTA + 10 ml/1.5 ml Iniprol ($10^6$ I.U.) (Choay Laboratory, 75782 Paris Cedex 16, France) from the marginal vein of the ear at 9.00 a.m.

The intravenous glucose tolerance test (IVGTT) was carried out at 180, 270 and 450 days on 8 C and 10 PDL rabbits. A glucose load was administered to the restrained rabbits by one i.v. injection of glucose 0.5 g/kg into the marginal vein of the ear. Blood was sampled from the other ear, 2 min before the injection of glucose (time 0) and 5, 30, 60 and 90 min thereafter.

Insulin and glucagon pancreatic stores were determined at 30 and 450 days. Four C and eight PDL animals per period were killed quickly and bled. Pancreatic tissue was minced in 10 volumes of an absolute alcohol/HCl/distilled water solution (250/5/78 v/v). The pancreas was crushed at 4°C in an Ultra-Turrax apparatus

(10,000 rpm for 2 min) followed by 10 strokes (20 sec each) in a Teflon glass Potter-type homogenizer and maintained at 4°C for four days then centrifuged (5000 rpm for 15 min). The supernatant was used at a final dilution of 1/5000 in 0.1 M PBS buffer, pH 7.4.

### Biochemistry

Plasma glucose was measured by an enzymatic method (glucose oxidase-GOD-PAP, Boehringer Mannheim, 38240 Meylan Cedex, France) in a COBAS-BIO autoanalyzer.

Immunoreactive insulin from 100 ml of plasma was determined by RIA using the SB-INSI-5 assay kit (Cis bio International, Oris Industry Co, 91192 Gif sur Yvette cedex, France), suitable for humans and animals, showing a weak reaction with proinsulin (3%). The detection limit was given as 2.50 ± 0.27 mU/ml by the manufacturer.

Plasma immunoreactive glucagon was measured by RIA according to the technique of Kervran *et al.*[22] using goat GAN antiserum (diluted 1/5000) and monoiodinated ($^{125}$I-labeled) glucagon (generous gifts from Pr. A. Kervran, Montpellier, France). GAN antiserum incubated for five days at 4°C recognized the −COOH terminal of pancreatic glucagon. Samples of 100 ml were used for each determination with 250 μl dextran–charcoal suspension, after 15 min of magnetic agitation at 4°C, 2 ml phosphate buffer 0.05 M were added to each tube. The radioactive precipitate was obtained after separation by centrifugation (6 min at 3200 rpm at 4°C) and counted in a gamma Counter 5000 (Packard Instr. Paris, France). The experimental detection limit reported for the technique was < 0.7 fmol/500 ml.

### Statistical Methods

The data were expressed as means ± SEM and compared by ANOVA and Student's unpaired and paired *t* tests. Differences were considered significant at $p < 0.05$. For the determination of hormonal insulin, pancreatic glucagon stores and IVGTT, data from C rabbits were pooled, since no differences were observed at the different time points.

### Immunocytochemistry

Samples from the pancreas (tail) of 27 rabbits (4 C and 5 PDL per period), used for the biochemical determinations at 180, 270 and 450 days, were processed as described elsewhere.[14]

### RESULTS

Glucose, insulin and glucagon were determined in the plasma of both control and PDL rabbits over a period of 450 days after operation. The glucose concentrations in the C group (Fig. 1a) ranged from 8.1 ± 0.7 mmol/l to 10.2 ± 0.8 mmol/l throughout the experiment. In the PDL group, two transient periods of significantly high values were noted, one at 15–90 days ($p < 0.01$), and the other at 450 days ($p < 0.02$).

Insulin levels (Fig. 1b) were significantly reduced on the 30th day (41.8 ± 7.6 pmol/l, $p < 0.01$) and the 180th day (83.8 ± 3.1 pmol/l, $p < 0.02$) compared to the control animals whose insulin profile progressed with alternating peaks.

In the C group, glucagon concentrations were 158 ± 16 ng/l throughout the experiment (Fig. 1c). After PDL, marked variations were observed with two distinct peaks: the first at 30 days (232 ± 21 ng/l, $p < 0.01$) and the second at 390 to 450 days (225 ± 25 ng/l, $p < 0.01$ and 199 ± 20 ng/l $p < 0.01$, respectively), separated by troughs that were below levels in the C group ($p < 0.05$) at 120 days (132 ± 7 ng/l) and 180 days (120 ± 3 ng/l).

From days 0 to 120, plasma glucagon and glucose were significantly correlated ($r = 0.91$, $p < 0.001$), whereas in the late hyperglucagonemic period, glucose release was elevated only at the 390th day, *i.e.*, 50 days later than glucagon. Up to 60 days, negative correlations were observed between circulating insulin and glucagon ($r = -0.95$, $p < 0.001$), and between insulin and

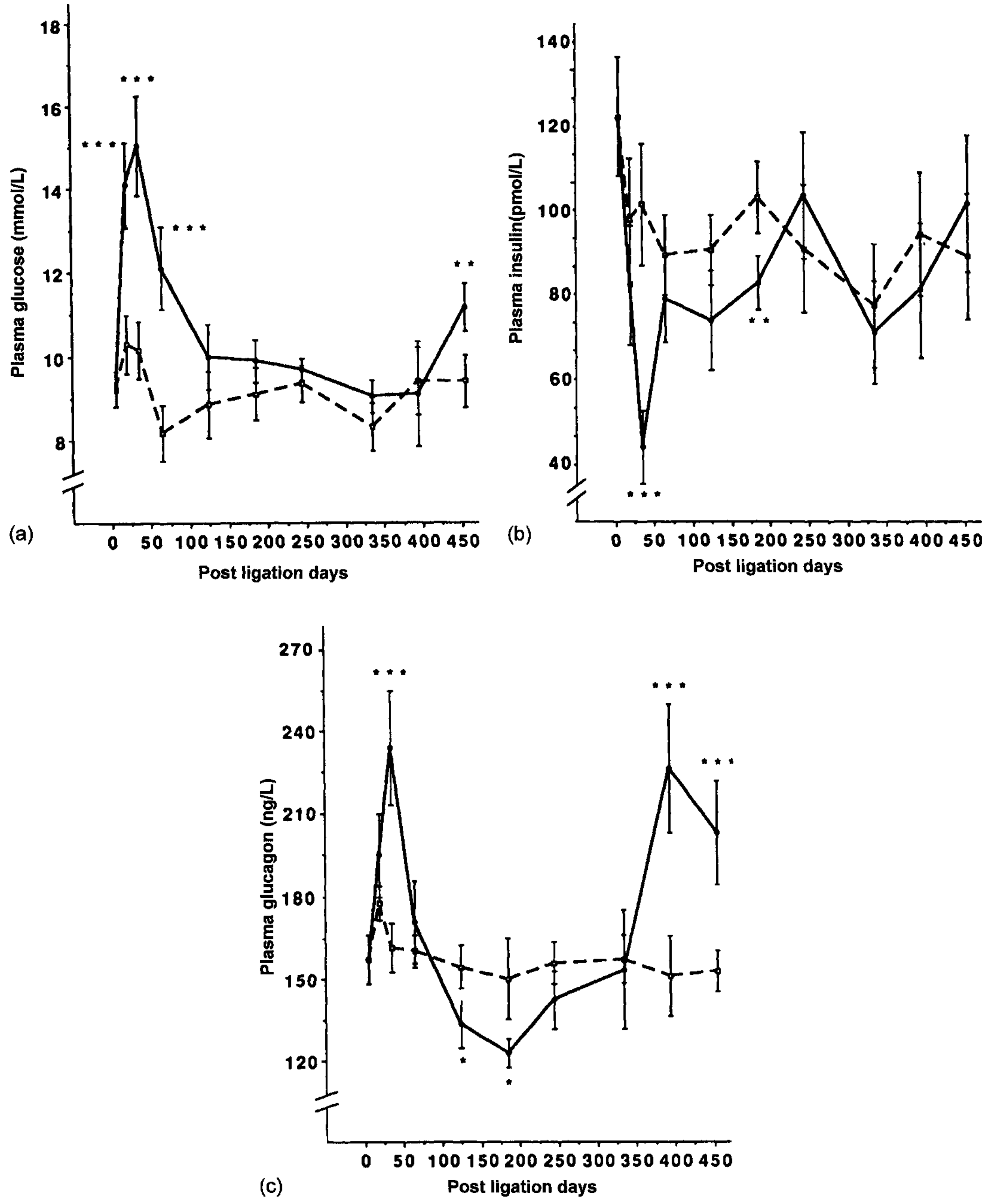

FIGURE 1 Comparative study of the concentrations of (a) plasma glucose (b) plasma insulin and (c) plasma glucagon in C rabbits (----) and P rabbits (——) from 0 to 450 days post-ligation. Each point indicats the mean ± SEM values for 8 C and 22 to 8 PDL animals. *p < 0.05, **p < 0.02 and ***p < 0.01.

glucose ($r = -0.94$, $p < 0.001$), but there was a positive correlation between glucose and glucagon ($r = 0.95$, $p < 0.001$).

The evolution of pancreatic insulin and glucagon stores is illustrated in Figure 2. In the PDL group, insulin had decreased

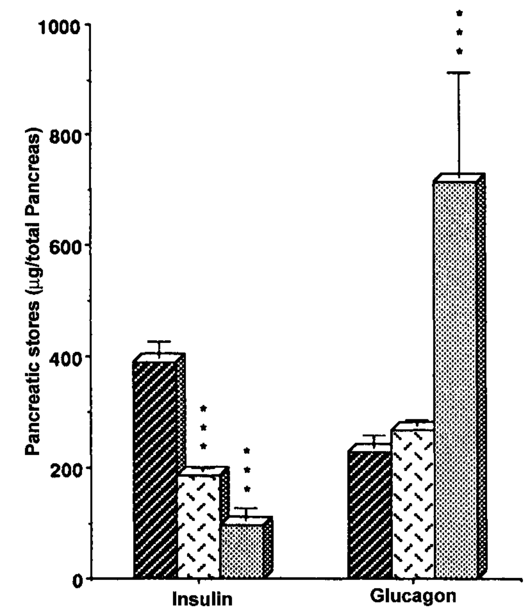

FIGURE 2 Comparison of insulin and glucagon concentrations in pancreatic gland in C rabbits (▨) and P rabbits (30 days ⧅ – 450 days ▩). Values given are mean ± SEM for 4 C to 8 PDL animals. ***p < 0.01.

2-fold at 30 days ($p < 0.01$) and 4-fold at 450 days ($p < 0.01$), while glucagon was 3-fold higher ($p < 0.01$) than in the C group at 450 days.

After the IVGTT, plasma glucose in the C group reached a peak at the 5th min then returned to basal values 60 min later. At 180, 270 and 450 days, the PDL rabbits exhibited the same profile, but with significantly higher values up to 60 min (from $p < 0.05$ to $p < 0.02$) (Fig. 3). Five min after injection, the insulin responses to the IVGTT of both groups (Fig. 4) reached a peak then returned to basal values at 90 min. The peak secretion in the PDL group was reduced 2-fold at 180 days ($p < 0.01$) and 5-fold at 270–450 days ($p < 0.01$). In the C group, glucagon had fallen to 50% of its initial level at 60 min, and then rose above basal levels at 90 min (Fig. 5). In the PDL group, glucagon was significantly elevated at 180

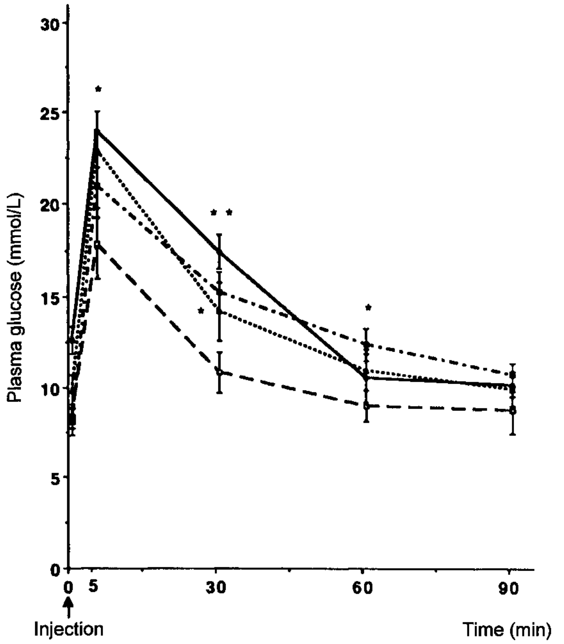

FIGURE 3 Intravenous glucose loading test (0.05 g/kg). Changes in plasma glucose response in C rabbits (– – –) and P rabbits at 180 (··········), 270 (—·—·—·) and 450 (———) days post ligation. Each curve plots means ± SEM for 8 C to 10 animals. *p < 0.05 and **p < 0.02.

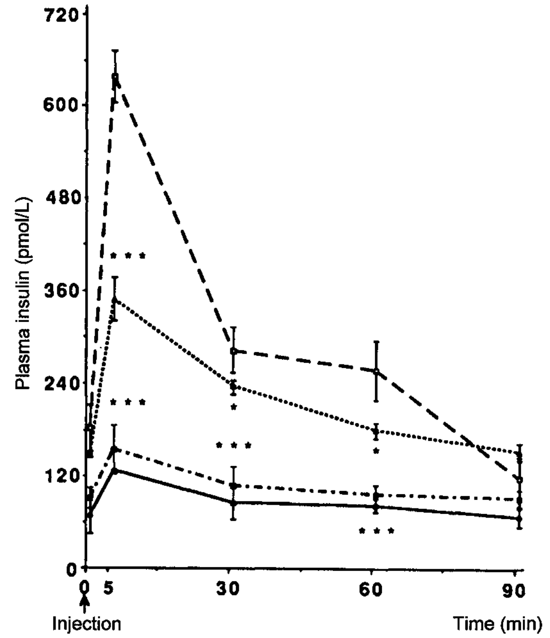

FIGURE 4 Intravenous glucose loading test (0.05 g/kg). Changes in plasma insulin response in C rabbits (– – – ·) and P rabbits at 180 (··········), 270 (—·—·—·) and 450 (———) days post -ligation. Each curve plots means ± SEM for 8 C to 10 PDL animals. *p < 0.05 and ***p < 0.01.

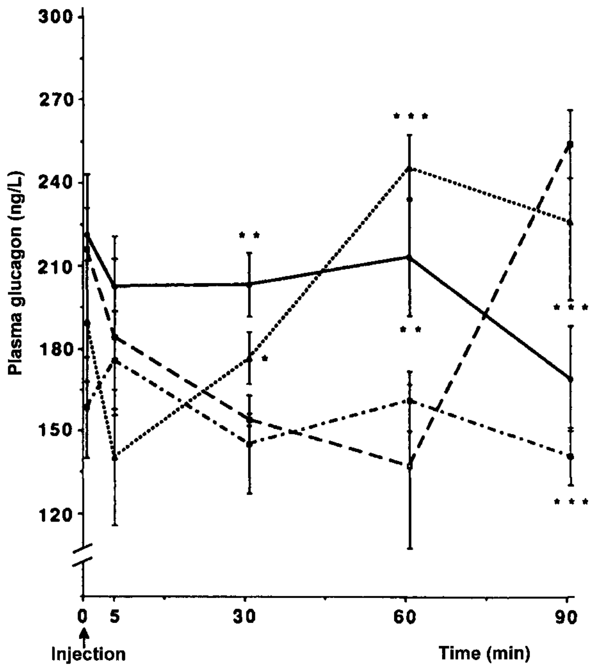

FIGURE 5 Intravenous glucose loading test (0.05 g/kg). Changes in plasma glucagon response in C rabbits (- - - -) and P rabbits at 180 (·········), 270 (—·—·—) and 450 (———) days post -ligation. Each curve plots means ± SEM for 8 C to 10 animals. *p < 0.05 and ***p < 0.01.

days by the 30th ($p < 0.05$) and 60th min ($p < 0.01$), whereas at 270 and 450 days, it remained unchanged at all times after injection of glucose.

The immunocytochemical observations on A-cells are illustrated in Figure 6. In the C group, immunostained cells for glucagon were found mainly on the periphery of the islets (Fig. 6a). After PDL, the marked A-cell immunoreactivity enabled us to localize the numerous changes in the long-term recasting pancreas. At 180 days (Fig. 6b), some of the A-cells were located irregularly around clusters of unstained endocrine cells and scattered among the fibro-connective tissue. At 270 days, a number of them were arranged in a discontinuous thick mantle encircling the clusters (Fig. 6c). Enlarged homologous and heterologous A-cell areas were distributed inside the fibrous tissue, which was itself surrounded by adipose tissue at 450 days (Fig. 6d).

### DISCUSSION

Pancreatic duct ligation (PDL) in our rabbits induced atrophy and fibrotic replacement of the pancreatic acini,[12] consistent with most of the effects produced by either duct ligation or occlusion in various species.[23–25] However, its influence on the endocrine system was less in accord with previous reports.[26,27] To our knowledge, the rabbit is the only laboratory animal that possesses a single pancreatic duct, separated by 40 cm from the bile duct.[28] Its ligation leads to neither severe nutritional disorders nor obesity,[29] which may be accounted for by compensatory digestive processes induced by microorganisms[30] or intestinal mucosal enzyme activities, which are thought to operate after PDL,[31] in patients with cystic fibrosis[32] and those with chronic pancreatic insufficiency associated with diabetes mellitus.[33]

In fact in rabbits, long-term pancreatic duct ligation induces no visible pathological signs, apart from a chronic and progressive diabetogenic state leading to irreversible pathology. However, our findings differed from those of Hepner *et al.*[34] who found blood glucose unchanged between 2 and 12 months after ligation in rabbits. This difference may be due to differences in breed and experimental conditions. In mini-pigs, pancreatic occlusion was not found to lead to glycemic disorders until 9 months,[35] whereas in rats, an increase in glycemia was described 5 to 8 months after ligation.[36]

The comparative variations in glycemia and insulinemia of the C and PDL rabbits are illustrated in Figures 1a,b. During the 1st month, the elevated glucose levels in the C group could be explained by the effects of the anesthesia and laparotomy followed by operative stress, as described in fasting sham rats.[37] The hyperglycemia in the PDL group was more marked

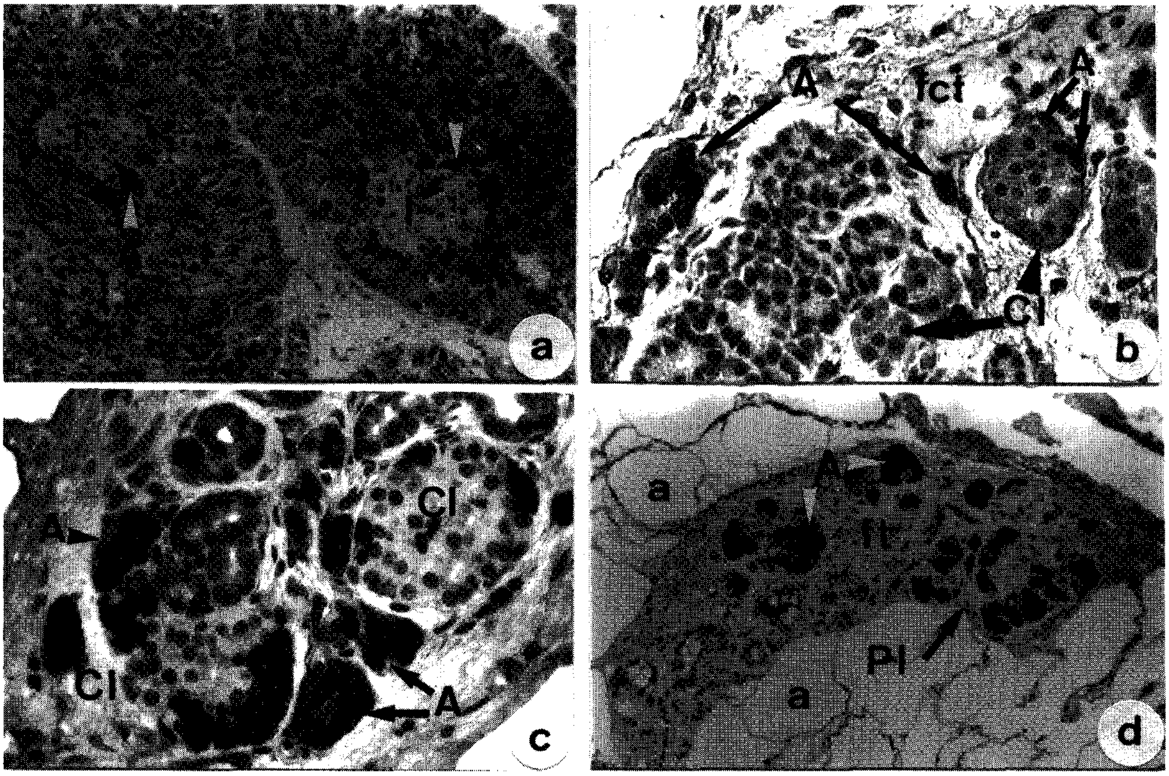

FIGURE 6 Long-term immunoreactive A cells in endocrine pancreas of control and pancreatic duct ligated rabbits. (a). *Controls*, A cells (A) are placed essentially at the periphery and sometimes inside the islet of Langerhans (I). (Magnification × 192). (b). 180 *days*, few A cells (A) are particularly well developed at the periphery of clusters (cl) or scattered among a fibroconnective tissue (fct). (Magnification × 480). (c). 270 *days*, maximum immunoreactivity of A cells (A) laid out in a thick mantle at the periphery of non-stained B cell clusters (cl). (Magnification × 480). (d). 450 *days*, intense immunoreactivity of numerous homologous A cell clusters (A) inside a fibrous tissue (ft) and around pseudo-islets (PI) surrounded by numerous adipocytes (a). (Magnification × 192).

than that found in previous data[11] with a maximum (15.1 ± 1.2 mmol/l) that correlated ($r = -0.94$, $p < 0.001$) with the nadir in insulin levels (42 ± 8 pmol/l) on day 30 (Fig. 1b). The plasma insulin and the insulin pancreatic store (200 ± 7 μg/total pancreas) (Fig. 2) were around half the levels in the C group (103 ± 13 pmol/l plasma insulin, and 400 ± 15 μg/total pancreas weight). Such reductions in plasma insulin and pancreatic insulin content have been described in perinatal STZ rats[38] and GK rats[20] and are thought to be the primary event in the progression of diabetes in NIDDM patients[39] and NIDDM GK rats.[1] After PDL, the transient hypoinsulinemic state is concomitant with a significant decline (−75%) in number of B-cells by the 30th day.[14] During the first month post-ligation, fibrosis was responsible for the dissociation of islets and the scattering of insular cells (B, A and D), leading to B-cell necrosis and partial degranulation[11] with a reduction in nuclear and cytoplasmic areas.[14] It was clear that the reduction in B-cell mass led to the hypoinsulinemia and the loss of insulin stores indicating that the regenerated B-cells became mature later.

From the 90th day, in the PDL group, glucose returned to its basal level, which was slightly higher than in the C group (Fig. 1a). At the same time, the basal plasma insulin did not differ significantly from that in the C group (Fig. 1b), which was indicative of regeneration of B-cells and their ability to secrete insulin. However, the observed insulinemia might have stemmed from the accumulation of large amounts of proinsulin, which has been found associated with the insulin defects in diabetics.[40] After PDL, during regeneration of B-cells, proinsulin may not be fully cleaved into active insulin. Moreover, as the ability of the basal plasma insulin to maintain euglycemia from the 90th day was time-limited, the available insulin was unable to prevent the later period of hyperglycemia (450 days). The plasma glucose increased by about 25% with respect to that observed in the C group, and rose continuously until 540 days (unpublished observations). At 450 days, the four-fold less insulin in pancreatic stores in the PDL group ($101 \pm 12\,\mu g$ *vs.* $400 \pm 15\,\mu g$/ total pancreas in the C group) (Fig. 2) indicated an inability of the regenerated B-cells to restore the initial pancreatic store giving rise to the long-term insulin impairment.

The changes in plasma glucagon levels throw light on other aspects of the glycemic defects after PDL (Fig. 1c). The basal plasma glucagon of $158 \pm 15$ ng/l in the C group remained relatively stable over 450 days. It ranges from 120 pg/ml in humans[18] and 180 pg/ml in dogs[41] to 323 pg/ml and 331 pg/ml in Wistar and GK rats, respectively (1). In contrast, our rabbits exhibited two significant hyperglucagonemic states (Fig. 1c) after PDL. The first, from 15 to 60 days appeared with a peak ($232 \pm 21.0$ ng/l, $p < 0.01$) at day 30. Immunocytochemical observations[14] have shown that as the B-cells are destroyed by the 30th day, the A-cells are preserved with hypertrophy of their nuclear areas, which is a reflection of hyperactivity. Moreover, the lack of alteration in pancreatic glucagon stores (Fig. 2) indicated that the A-cells released rather than stored glucagon. Their hyperactivity was probably the consequence of a loss of glucagon-inhibiting action from the dispersal of B-and D-cells in fibrotic tissue.[42,43] A hyperglucagonemic state had been reported in NIDDM patients,[44,45] in diabetic rats,[46] dogs,[47] and db/db mice.[48] In contrast to fetuses, adult GK rats[1] and neonatal STZ diabetic rats,[38] basal plasma glucagon was found to be unchanged and even lower in mini-pigs after pancreatic occlusion.[35] The second hyperglucagonemic state (Fig. 1c) between 390 days ($225 \pm 25.4$ ng/l, $p < 0.01$) and 450 days ($199 \pm 20$ ng/l, $p < 0.01$) being about 50% higher than the levels in the C group, was consistent with the morphological changes in the regenerated cells illustrated in Figure 6. There was an increase in number of immunostained A-cells, which formed a thick crown surrounding the clusters of B-cells called pseudo-islets.[14] These features were most marked from 270 to 450 days (Figs. 6c,d) and probably caused the increase in both glucagonemia and pancreatic glucagon content (3-fold at 450 days) (Fig. 2). The ability of the A-cells to continuously synthesize glucagon by releasing and storing it, showed that the regenerated insular A-cells had escaped normal endocrine regulation in the pseudo-islets. The simultaneous decrease in B-cells and increase in A-cells after PDL were similar to the evolution of pancreatic islet cells in either spontaneous[49] or induced[50] type 2 diabetes. In our experimental model, these events could be attributed to glucagonoma accounting for the uncontrolled glucagon release giving rise to an irreversible diabetogenic effect, as noted in the course of NIDDM in man.[51]

When comparing the long-term (from 0 to 450 days) plasma glucagon profile (Fig. 1c) with glucose levels (Fig. 1a), the insulinemia (Fig. 1b) was taken into account. From 0 to 120 days, there was a significant correlation between glucose and glucagon levels ($r = 0.91$, $p < 0.001$) corresponding to the hyperglycemic and insulinopenic episodes. Up to 60 days, there was a positive correlation ($r = 0.95$, $p < 0.001$) between plasma glucose and glucagon, but a negative correlation between glucose and insulin ($r = -0.94$, $p < 0.001$), and between insulin and glucagon ($r = -0.95$, $p < 0.01$). By contrast, in STZ diabetic rats, there is a concomitant hyperglucagonemia and hyperglycemia with a delay in the insulinopenia.[52] After PDL, it was not clear which hormone was responsible for the first hyperglycemic period, although the combined effects of hypoinsulinemia and hyperglucagonemia enhance HGO. In diabetic patients, hyperglucagonemia involves a primary insulinopenia whose action was on gluconeogenesis rather than glycogenolysis[53] leading to excessive hepatic glucose production.[54]

There was another possible relationship between the second hyperglucagonemic episode (Fig. 1c) at 340–450 days and the hyperglycemic one starting 50 days later (Fig. 1a), without hypoinsulinemia (Fig. 1b). In fact, glucose was released to a lesser extent and was delayed with respect to glucagon, whereas glucagon levels were identical to those of the first. A new regulation of the glucagon response may exist in the HGO when the plasma insulin had returned to its basal level producing its inhibiting effects. These observations would suggest that PDL did not lead to long-term insulin resistance. Despite the hyperglucagonemia, the indirect action of insulin in suppressing endogenous glucose production is considered to be a dominant event in human type 2 diabetes[55] and STZ rats.[56]

The opposite actions of glucagon (Fig. 1c) and insulin (Fig. 1b) in maintaining euglycemia between the two hyperglycemic phases (Fig. 1a) from 120 to 390 days was indicative of a certain stability and functionality of the regenerated islets cells during a limited post-ligation period.

However, the IVGTT data demonstrated aggravated intolerance to glucose in the long-term after PDL despite the presence of basal plasma insulin levels. At 180, 270 and 450 days, 5, 30, and 60 min after glucose injection, plasma glucose levels remained significantly higher than those measured in the C group (Fig. 3). At 5 min, the insulin response appeared to decline markedly at 180 and 270–450 days (2-fold to 5-fold, respectively) (Fig. 4), corroborating the impaired insulin responses observed at 5, 15 and 30 days by previous data.[11] This has been typically linked with NIDDM and FCPD diseases in humans,[18] rats (57–58), and with chronic pancreatitis.[3] The progressive reduction in insulin response after PDL is consistent with the immunocytological findings (Fig. 6). At 180 days post-ligation (Fig. 6b), when the insulin response was least reduced (2-fold), we noted some clusters of unstained B-cells with a few stained A-cells leading an aspect similar to the original islets. However, the markedly impaired insulin release observed at 270 days, and especially at 450 days (Fig. 4) illustrated the insensitivity of B-cells in the pseudo-islets accompanied by a decrease in pancreatic insulin stores (about 4-fold *vs.* controls) (Fig. 2). These observations indicated that the regenerated B-cell mass was unable to synthesize active insulin in response to stimulation and could not even maintain pancreatic stores (Fig. 1b). This deficit in insulin secretion in regenerated B-cells has also been described in Zucker diabetic fatty (ZDF)[59] and STZ rats.[60]

As the glucagon response to the IVGTT in rabbits has not yet been described, these experiments (Fig. 5) supplied further information about A-cell function in the regenerated islets greatly enriched by A-cells (Figs. 6c,d). In the C group, the glucagon profile was similar to that of humans[18] *i.e.*, with an initial progressive fall up to 60 min followed by a significant increase at 90 min (Fig. 5). After PDL (Fig. 5), by 180 days, the plasma glucagon response declined at 5 min as in controls, but there was a significant increase from 30 min ($p < 0.05$) to 60 min ($p < 0.01$). The same hyperglucagonemic profile has been described in diabetic dogs[41] after oral glucose administration. After PDL, on appearance of new islets near the original structure (Fig. 6b), the glucose sensitivity of both A- and B-cells was found to be less affected than at 270–450 days (Figs. 6c,d). At these later periods, the nearly horizontal profile (Fig. 5) indicated that the plasma glucagon response was abolished, demonstrating the insensitivity of A-cells to glucose. These results showed that the regenerated islets with the numerous A-cells associated with an elevated (3-fold) pancreatic glucagon content were unable to reduce production of glucagon. The horizontal glucagon profile in response to

IVGTT after PDL was comparable to that found in tropical FCPD[18] and NIDDM patients[61] as well as in chronic pancreatitis.[8]

These experiments demonstrated that in rabbits, abnormalities in hormonal responses of islet cell due to alterations inherent in the reconstitution of pseudo-islet cells were comparable to the impaired hormone responses in NIDDM patients.[62]

The defects after PDL mimicked the main features of the NIDDM syndrome, FCPD and pancreatitis in both humans and animals, and throw more light on the relationships between the antagonistic pancreatic hormones in the regulation of blood glucose. The PDL rabbit would thus appear to be a suitable experimental model for further investigations on the pathophysiology and treatment of pancreatic disorders.

### *References*

[1] Movassat, J., Saulnier, C., Serradas, P. and Portha, B. (1997). Impaired development of pancreatic beta-cell mass is a primary event during the progression to diabetes in the GK rat. *Diabetologia*, **40**(8), 916–925.
[2] Iwashima, Y., Watanabe, K. and Makino, I. (1990). Changes in the pancreatic A-, B- and D-cell populations during development of diabetes in spontaneously diabetic chinese hamsters of the Asahikawa colony (CHAD). *Diabetes Res. Clin. Pract.*, **8**, 201–214.
[3] Iakhontova, O. I. and Pomazouskaia, V. A. (1991). Hormonal mechanism of carbohydrate metabolism disorders in chronic pancreatitis. *Ter. Arkh.*, **63**(2), 55–58.
[4] Yajnick, C. S., Shelgikar, K. M. *et al.* (1992). The ketosis-resistance in fibro-calculous pancreatic diabetes-1. Clinical observations and endocrine metabolic measurements during oral glucose tolerance test. *Diabetes Res. Clin. Pract.*, **15**(2), 149–156.
[5] De Fronzo, R. A. (1992). Pathogenesis of type II (non-insulin-dependent) diabetes mellitus: a balanced over view. *Diabetologia*, **35**, 389–397.
[6] Brand, C. L., Rolin *et al.* (1994). Immunoneutralization of endogenous glucagon with monoclonal glucagon antibody normalizes hyperglycemia in moderately streptozotocin diabetic rats. *Diabetologia*, **37**(10), 985–993.
[7] Tasaka, Y., Inoue, Y., Matsumoto, H. and Hirata, Y. (1988). Changes in plasma glucagon, pancreatic polypeptide and insulin during development of alloxan diabetes mellitus in dog. *Endocrinol. Jpn.*, **35**(3), 399–404.
[8] Kannan, V., Nabarro, J. D. and Cotton, P. B. (1979). Glucagon secretion in chronic pancreatitis. *Horm. Res.*, **11**, 203–212.
[9] Consoli, A. (1992). Role of liver in pathophysiology of NIDDM. *Diabetes care*, **15**, 430–441.
[10] Samols, E., Bonneir-Weir, S. and Weir, G. C. (1986). Intra-islet insulin-glucagon-somatostatin relationship. *Clinics in Endocrinology and Metabolism*, **15** (1), 33–58.
[11] Catala, J., Bonnafous, R. and Hollande, E. (1986). Disturbances in the regulation of glycaemia in rabbits following PDL. Biochemical and immunocytochemical studies. *Diabète Métab.*, **12**, 203–211.
[12] Catala, J., Bonnafous, R., Dutrillaux, M. C. and Hollande, E. (1987). Dissociation of Langerhans islets in the rabbit after PDL. Immunocytochemical and ultrastructural studies. *Virchows Arch. (cell pathol)*, **52**, 539–551.
[13] Petkov, P., Donev, S., Catala, J., Bonnafous, R., Fichaux, F. and Desbals, B. (1992). Evolution of the pancreatic parenchyma after duct ligature: a histological and ultrastructural study. *Zool. Jb. Anat.*, **122**, 1–16.
[14] Cortie, C., Catala, J., Bonnafous, R. and Petkov, P. (1992). Immunocytochemistry and morphometry of insular A, B and D cells in rabbit during long-term pancreatic duct ligation. In: *Recent advances in cellular and molecular biology*, Wegmann R. J. and Wegmann M. A., Eds. Paris, **5**, 195–207.
[15] Catala, J., Hadjiisky, P., Bonnafous, R. and Fichaux, F. (1990). Cell features in pancreas of prediabetic and diabetic rabbits after Wirsung duct ligation. Histochemical and histoenzymatic studies. *Acta diabetol. lat.*, **27**, 59–69.
[16] Fichaux, F., Catala, J. and Bonnafous, R. (1992). Responsiveness and memory of the pancreatic B-cells to the insulin secretagogues D-glucose and L- arginine in prediabetic and diabetic rabbits. *Pancreas*, **5**, 585–594.
[17] Poitout, V. and Robertson, R. P. (1996). An integrated view of beta-cell dysfunction in type II diabetes. *Ann. Rev. Med.*, **47**, 69–83.
[18] Mohan, V., Snehalatha, C., Ramachandran, A., Chari, S., Madanagopalan, N. and Viswanathan, M. (1990). Plasma glucagon responses in tropical fibrocalculous pancreatic diabetes. *Diabetes Res. Clin. Pract.*, **9**, 97–101.
[19] Solomon, S. S., Duckworth, W. C., Jallepalli, P., Bobal, M. and Lyer, R. (1980). The glucose intolerance of acute pancreatitis. Hormonal response to arginine. *Diabetes*, **29**, 22–26.
[20] Portha, B., Giroix, M. H., Serradas, P., Morin, L., Tormo, M. A. and Bailbé, D. (1994). Cellular basis of pancreatic B cells in non-insulin-dependent diabetes. In: *Insulin secretion and pancreatic B-cell research*. Smith, G., Flah, P. R. and Leuzen S. (Eds.), London, pp. 461–472.
[21] Catala, J. (1976). Contrôle de l'efficacité de la ligature du canal pancréatique chez le lapin. *Ann. Rech. Vétér.*, **7**, 33–38.
[22] Kervran, A., Blache, P. and Bataille, D. (1987). Distribution of oxyntomodulin and glucagon in the gastrointestinal tract and the plasma of the rat. *Endocrinology*, **121**, 704–713.
[23] Little, J. M., Lauer, C. and Hogg, J. (1977). Pancreatic duct obstruction with an acrylate glue: a new method for producing pancreatic atrophy. *Surgery St Louis*, **81**, 243–249.
[24] Gebhardt, C. and Stolte, M. (1978). Pancreasgeng Okklusion durch injection einer schnell härtenden amino-saürenlösung. *Arch. Klin. Chir.*, **346**, 149–166.
[25] Metz, J., Merlo, M., Billich, H. and Forssmann, W. G. (1978). Exocrine pancreas under experimental conditions. Alteration of intercellular junction between acinar cells following pancreatic duct ligation. *Cell. Tis. Res.*, **186**, 227–240.

[26] Boquist, L. and Edstrom, C. (1970). Ultrastructure of pancreatic acinar and islet parenchyma in rats at various intervals after duct ligation. *Virchows Arch.*, **349**, 69–70.
[27] White, D. C., Sutherland, D. E. and Najarian, J. S. (1981). Endocrine function and histology of the canine pancreas after exocrine ablation by ductal injection of silicone rubber adhesive. *J. Surg. Res.*, **31**, 361–374.
[28] Brachet, A. (1935). In: *Traité d'embryologie des Vertébrés.* Masson Ed Paris
[29] Catala, J. (1976). Variations du comportement alimentaire de la croissance et de la digestibilité chez des lapins à canal pancréatique ligaturé. *Ann. Biol. Anim. Bioch. Biophys.*, **16**(5), 687–697.
[30] Catala, J. and Bonnafous, R. (1979). Variations de l'activité $\alpha$-amylasique parietale et intraluminale dans le tube digestif de lapin témoin et à canal pancréatique ligaturé. *Ann. Biol. Anim. Bioch. Biophys.*, **19**(3B), 813–817.
[31] Catala, J. and Bonnafous, R. (1982). Adaptation of the activities of the brush border hydrolases along the small intestine of the rabbit after pancreatic duct ligation. *Digestion*, **24**, 234–240.
[32] Antonowicz, I., Lebenthal, E. and Shwachman, H. (1978). Disaccharidase activities in small intestinal mucosa in patients with cystic fibrosis. *J. Pediatr.*, **92**, 214–219.
[33] Cerda, J. J., Reiser, H. and Crane, R. K. (1972). Brush border enzymes and mal absorption elevated disaccharidases in chronic pancreatic insufficiency with diabetes mellitus. *Gastroenterology*, **6**(2), 841.
[34] Heptner, W., Neubauer, H. P. and Schleyerbach, R. (1974). Glucose tolerance and insulin secretion in rabbits and dogs after ligation of the pancreatic ducts. *Diabetologia*, **10**, 193–196.
[35] Schwille, P. O., Engelhardt, W., Volkholz, H., Gebhardt, C., Zirngibl, H. and Stolte, M. (1985). Influence of pancreatic duct occlusion on islet hormones in peripheral and portal plasma in the pancreas of the mini-pig. *Eur. Surg. Res.*, **17**, 17–24.
[36] Edstrom, C. and Falkmer, S. (1968). Pancreatic mophological and blood glucose level in rats at various intervals after duct ligation. *Virchows Arch.*, **345**, 139–153.
[37] Yamada, F., Inque, S. *et al.* (1993). Glucoregulatory hormones in the immobilization, stress-induced increase of plasma glucose in fasted and fed rats. *Endocrinology*, **132**(5), 2199–2205.
[38] Portha, B., Levacher, C., Picon, L. and Rosselin, G. (1974). Diabetogenic effect of streptozotocin in the rat during the perinatal period. *Diabetes*, **23** (11), 889–895.
[39] Klöppel, G., Löhr, M., Hablich, K., Oberholzer, M. and Heitz P. U. (1985). Islet pathology and pathogenesis of type I and type II diabetes revisited. *Surv. Synth. Path. Res.*, **4**, 110–125.
[40] Polonsky, K. S. (1999). Evolution of beta-cell dysfunction in impaired glucose tolerance and diabetes. *Exp. and clin. Endocrinol. and diabetes*, **107** suppl. (4), 124–127.
[41] Hillaire-Buys, D., Ribes, G., Gross, R. and Loubatières-Mariani, M. M. (1987). Paradoxical hyperglucagonemia after oral administration of glucose in diabetic dogs. Impact of the cholinergic nervous system. *CR Soc. Biol. Fil.*, **181**, 677–682.
[42] Kaneko, K., Shirotani, T., Araki, E., Matsumoto, K., Taguchi, T., Motoshima, H., Yoshizato, K., Kishikawa, H. and Shichiri, M. (1999). Insulin inhibits glucagon secretion by the activation of PI3 kinase in In-R1-G9 cells. *Diabetes Res. & Clin. Pract.*, **44**(2), 83–92.
[43] Strowski, M. Z., Parmar, R. M., Blake, A. D. and Schaeffer, J. M. (2000). Somatostatin inhibits insulin and glucagon secretion *via* two receptors subtypes: an *in vitro* study of pancreatic islets from somatostatin receptor 2 knockout mice. *Endocrinology*, **141**(1), 111–117.
[44] Reaven, G. M., Chen, Y. D., Golay, A., Swislocki, A. L. and Jaspan, J. B. (1987). Documentation of hyperglucagonemia throughout the day in nonobese and obese patient with non-insulin-dependent diabetic subjects. *J. Clin. Endocrinol. Metab.*, **64**, 106–110.
[45] Walczak, M., Mrozikiewicz, D., Dmochowski, K., Rewers, M. and Cichy, W. (1989). Serum pancreatic polypèptide and glucagon immunoreactivity in fasting healthy and diabetic children. *Mater. Med. Pol.*, **21**, 38–42.
[46] Tomita, T., Sasaki, S., Doull, V., Bunag, R. and Kimmel, J. R. (1986). Pancreatic hormones in streptozotocin diabetes rats. *Int. J. Pancreatol.*, **1**(3–4), 265–278.
[47] Tasaka, Y., Inoue, Y., Matsumoto, H. and Hirata, Y. (1988). Changes in plasma glucagon, pancreatic polypeptide and insulin during development of alloxan diabetes mellitus in dog. *Endocrinol. Jpn.*, **35**, 399–404.
[48] Kodama, H., Fujita, M. *et al.* (1994). The possible role of age-related increase in the plasma glucagon/insulin ratio in the enhanced hepatic gluconeogenesis and hyperglycaemia in genetically diabetes (c57 Bz/KsJ db/db mice). *Jpn J. Pharmacol.*, **66**(3), 281–28759.
[49] Clark, A., Wells, C. A., Buley, I. D., Cruickshank, J. K., Vanhegan, R. I., Matthews, D. R., Cooper, G. J., Holman, R. R. and Turner, R. C. (1988). Islet amyloid, increased A-cells, reduced B-cells and exocrine fibrosis: quantitative changes in the pancreas in type 2 diabetes. *Diabetes Res.*, **9**, 151–159.
[50] Iwo, K., Bellomo, S. C., Mukai, N. and Craighead, J. E. (1983). Encephalomyocarditis virus induced diabetes mellitus in mice: long-term changes in the structure and function of islets of Langherans. *Diabetologia*, **25**, 39–44.
[51] Warner, T. F., Block, M., Hafez, G. R., Mack, E., Lloyd, R. V. and Bloom, S. R.(1983). Glucagonomas. Ultrastructure and immunocytochemistry. *Cancer*, **51**(6), 1091–1096.
[52] Dubuc, P. U. (1987). Hormonal responses during development of streptozotocin diabetes in rats. *Endocrinol. Exp.*, **21**, 275–284.
[53] Girard, J. (1995). Rôle des acides gras libres dans l'insulinorésistance au cours du diabète non insulinodépendant. *Diabète Metab.*, **21**, 79–88.
[54] Picarel-Blanchot, F., Berthelier, C., Bailbé, D. and Portha, B. (1996). Impaired insulin secretion and excessive hepatic glucose production an both early events in the diabetic GK rat. *Am. J. Physiol.*, **34**, E755–E762.
[55] Mittleman, S. D., Fu, Y. Y., Rebrin, K., Steil, G. and Bergman, R. N. (1997). Indirect effects of insulin to suppress endogenous glucose production is dominant even with hyperglucagonemia. *J. Clin. Invest.*, **100**(12), 3121–3130.
[56] Burcelin, R., Eddouks, M., Maury, J., Kande, J., Assan, R. and Girard, J. (1995). Excessive glucose production, rather than insulin resistance, accounts for hyperglycemia in recent- onset streptozotocin diabetic rats. *Diabetologia*, **38**(3), 283–290.
[57] Portha, B., Picon, L. and Rosselin, G. (1979). Chemical diabetes in the adult rat as the spontaneous evolution of neonatal diabetes. *Diabetologia*, **17**, 371–377.
[58] Berthelier, C., Picarel, F., Kergoat, M. and Portha, B. (1995). Ageing glucose metabolism and insulin action in the diabetic GK rat. Comparison with the normal Wistar rat. *Diabetologia* suppl. 1, **38** - 530A (Abstract).

[59] Pick, A., Clark, J., Kubstruq, C., Levisetti, M., Pugh, W., Bonneir-Weir, S. and Polonsky, K. S. (1998). Role of apoptosis in failure of beta-cell mass compensation for insulin resistance and beta-cell defects in the male Zucker diabetic fatty rat. *Diabetes*, **47**(3), 358–364.

[60] Bernard, C., Thibault, C., Berthault, M. E., Magnan, C., Saulnier, C., Portha, B., Pralong, W. F., Penicaud, L. and Ktorza, A. (1998). Pancreatic beta-cell regeneration after 48h glucose infusion in mildly diabetic rats is not correlated with functional improvement. *Diabetes*, **47**(7), 1058–1065.

[61] Tangs, S. (1992). Changes in plasma glucagon concentration in patients with diabetes mellitus and its clinical significance. *Chung Hua Hsueh Tsa Chih*, **72**(4), 222–255.

[62] Mooradian, A. D., Albert, S. G., Bernbaum, M. and Plummer, S. (1996). The effect of glipizide gastrointestinal therapeutic system on islet cell hormonal responses to a test meal in NIDDM. *Diabetes Care*, **19**, 8.

